# Biomechanical properties of a novel cervical spine implant with elastic deformation: a cadaveric study

**DOI:** 10.3389/fbioe.2023.1214877

**Published:** 2023-08-29

**Authors:** Haimiti Abudouaini, Tingkui Wu, Yang Meng, Chen Ding, Hao Liu, Wang Beiyu

**Affiliations:** ^1^ Department of Orthopedic Surgery, West China Hospital, Sichuan University, Chengdu, China; ^2^ Department of Spine Surgery, Honghui Hospital, Xi’an Jiaotong University, Xi’an, China

**Keywords:** cervical implant, biomechanics, elastically deformable, CAGE, artificial cervical disc

## Abstract

**Introduction:** Anterior cervical discectomy and fusion (ACDF) is a most frequently used surgical procedure for treating cervical radiculopathy and myelopathy. However, there is concern about the high adjacent segment degeneration (ASD) rate after ACDF surgery. We creatively designed an elastically deformable cervical implant to reduce the postoperative stress concentration. In this study, we aimed to investigate the biomechanical performance of this novel cervical implant and compare it with the commonly used cervical devices.

**Methods:** Biomechanical test was conducted on twelve fresh-frozen human cadaveric cervical spines (C2–C7) and randomly divided into four groups according to implant types: intact group, Zero-P VA fusion (ACDF) group, the novel cervical implant group and Pretic-I artificial cervical disc (ACDR) group. An optical tracking system was used to evaluate the segmental range of motion (ROM) of the C4/C5, C5/C6, and C6/C7 segments and micro pressure sensor was used to record the maximum facet joint pressure (FJP), maximum intradiscal pressure (IDP) at the C4-5 and C6-7 segments.

**Results:** There were no significant differences in the ROM of adjacent segments between the groups. Compared with the intact group, the ACDR group essentially retained the ROM of the operated segment. The novel cervical implant decrease some ROM of the operated segment, but it was still significantly higher than in the fusion group; The maximum FJP and IDP at the adjacent segments in the ACDF group were significantly higher than those values in the other groups, and there were no differences in the other groups.

**Conclusion:** While the newly developed elastically deformable cervical implant does not completely maintain ROM like the artificial cervical disc, it surpasses the fusion device with regards to biomechanical attributes. After further refinement, this novel implant may be suitable for patients who are prone to severe adjacent segment degeneration after fusion surgery but no indication for artificial cervical disc surgery.

## Introduction

Cervical intervertebral disc degeneration (CIDD) is a common syndrome characterized by pathological changes in intervertebral discs and causes secondary damage to important surrounding tissues, such as the spinal cord and nerve roots (Badhiwala et al., 2020a; Tian et al., 2021). According to a cohort study involving 47,560 patients, the incidence of CIDD is 13.1% (Schairer et al., 2014), which is higher than the prevalence of diabetes (9.7%) (Yang et al., 2010). The vast majority of patients with CIDD can alleviate their symptoms by using systematic conservative treatments; however, a small number of patients still require surgical treatment.

The cervical intervertebral cage successfully mimics the function of a normal cervical disc by being able to support the height of the intervertebral disc, as well as withstanding the pressure transmitted from the head and upper cervical level and protecting the nerve roots and spinal cord. However, the main biomechanical changes after cervical fusion surgery include the concentration of stress and the compensatory increases in the ranges of motion in the upper and lower adjacent segments (Eck et al., 2002; Pu et al., 2014; Alhashash et al., 2018). These biomechanical changes may cause adjacent segment degeneration (ASD), the loss of disc height at the operated segment, the formation of pseudoarthosis, and the subsidence of the intervertebral cage (Matsunaga et al., 1999; Basques et al., 2019; Abudouaini et al., 2021). When compared with the cervical intervertebral fusion device, the artificial cervical disc prosthesis not only realizes the function of supporting and withstanding the pressure of the normal cervical disc of the human body but also achieves the function of normal cervical disc motion. Such postoperative biomechanical changes effectively delay the adjacent level degeneration caused by compensatory overactivity after surgery. Finite element studies and biomechanical analyses have confirmed that cervical arthroplasty devices preserve normal motion, disc stresses, and facet loading at the adjacent levels (Puttlitz et al., 2004; Zhao and Yuan, 2019a; Peng et al., 2022). However, the surgical indications for ACDR are particularly narrow. At present, although there are no unified criteria for the indications of ACDR, these criteria can be summarized as mild-to-moderate CIDD with certain degrees of preoperative range of motion (ROM) (Stieber et al., 2011a; Womack et al., 2011; Faizan et al., 2012; Zhao and Yuan, 2019b).

When the human cervical spine is in vigorous activity or impacted, the cervical intervertebral disc will undergo elastic deformation, distribute the load to the surrounding areas, absorb the concussion of the spine exerted by the external forces, and serve a stress-buffering role. Currently applied cervical intervertebral cages have not well-mimicked the resilience and cushioning performance of normal cervical discs, and it is difficult to effectively distribute the load, which can easily lead to stress concentrations. Elastic polymeric materials exhibit special absorbed compressional energy performance when subjected to mechanical impact and compression, which provides them with certain advantages in mimicking the cushioning and shock absorption functions of the normal intervertebral disc. When an elastic polymer material deforms, it is accompanied by energy input and output. Specifically, when the elastic polymer material begins to deform to the maximum limit of deformation, the process represents a continuous input of energy. In the process of recovering from the maximum limit to the predeformation process, a portion of the energy is released, whereas the other portion of the energy is internally converted from mechanical energy to other potential energy. Consequently, it is theoretically possible to reduce the stresses that are transmitted to adjacent segments after cervical surgery.

Thus, in 2015, we designed a novel cervical implant with enhanced cushioning and shock absorption functions combined with personalized morphology and obtained a national invention patent. The design concept of this new cervical implant allows for the provision of satisfactory stabilization. In addition, appropriate deformation can occur to dynamically adapt to the stress variation and can reduce the interfacial micromotions, which can thereby reduce adjacent segment stress and maintain the operated segment intervertebral height. Therefore, the purpose of this study was to observe the biomechanical properties of the novel implant and to compared it with existing cervical prostheses. The results of this study may provide a theoretical basis for further improvements of this novel cervical implant, as well as further animal studies and human clinical applications.

## Materials and methods

### Specimen preparation

**FIGURE 1:**
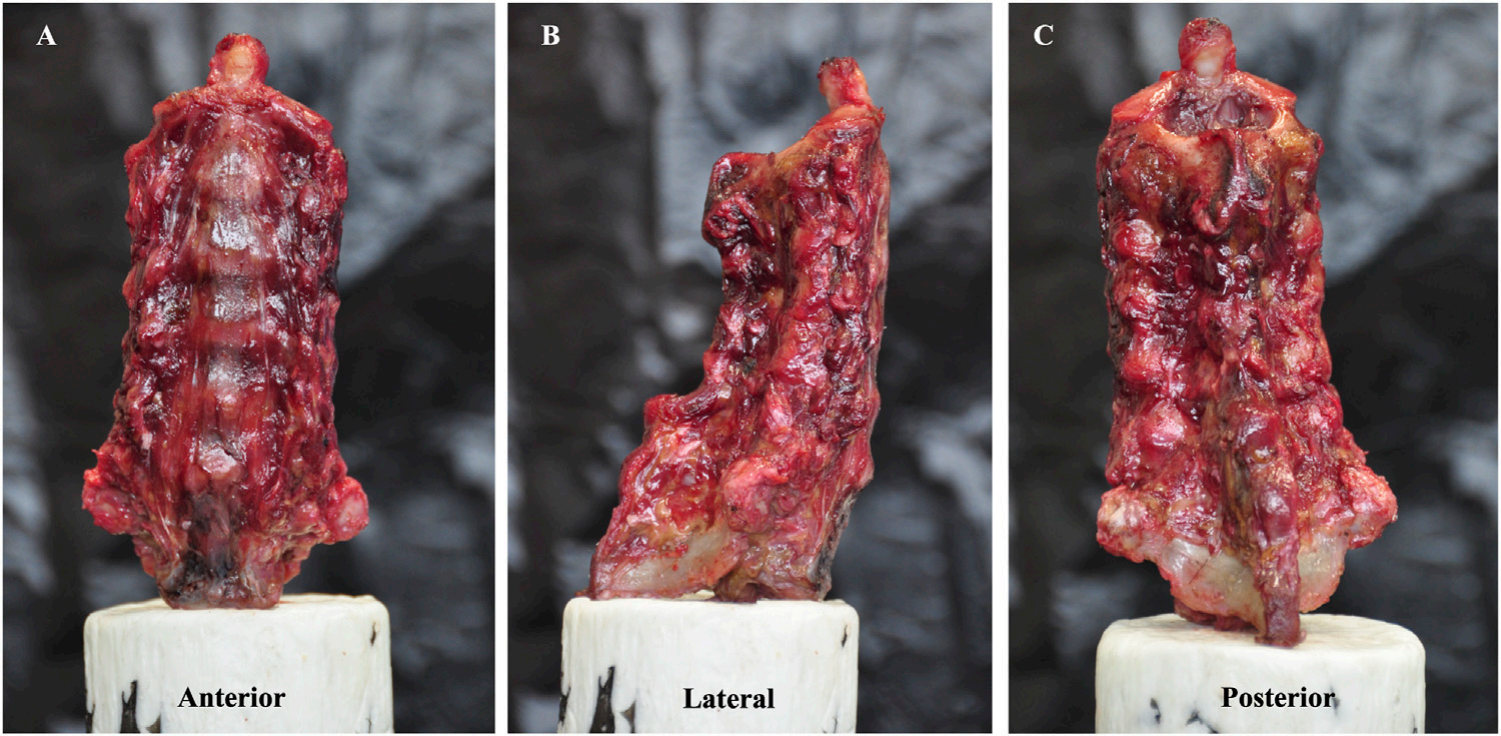
Specimen model of the C2–C7 cervical spine. **(A)** is the anterior view, **(B)** is the lateral view and **(C)** is the posterior view.

This study was an *in vitro* biomechanical study and was approved by the Institutional Review Board of West China Hospital. Twelve fresh-frozen human cadaveric cervical spines (C2–C7) that were taken from donors were included in this biomechanical test. Radiographs and bone scanning were performed to exclude specimens with obvious flaws, such as fractures, deformities, tumours, osteoporosis, or disc degeneration (including osteophytes, disc space narrowing, or facet hypertrophy). The surrounding soft tissues and muscles of the twelve specimens were carefully dissected. The ligamentous structures, intervertebral discs, and facet joint capsules were preserved. All of the specimens were placed in double plastic bags and stored at −20°C (Figure 1).

### Implants

**FIGURE 2:**
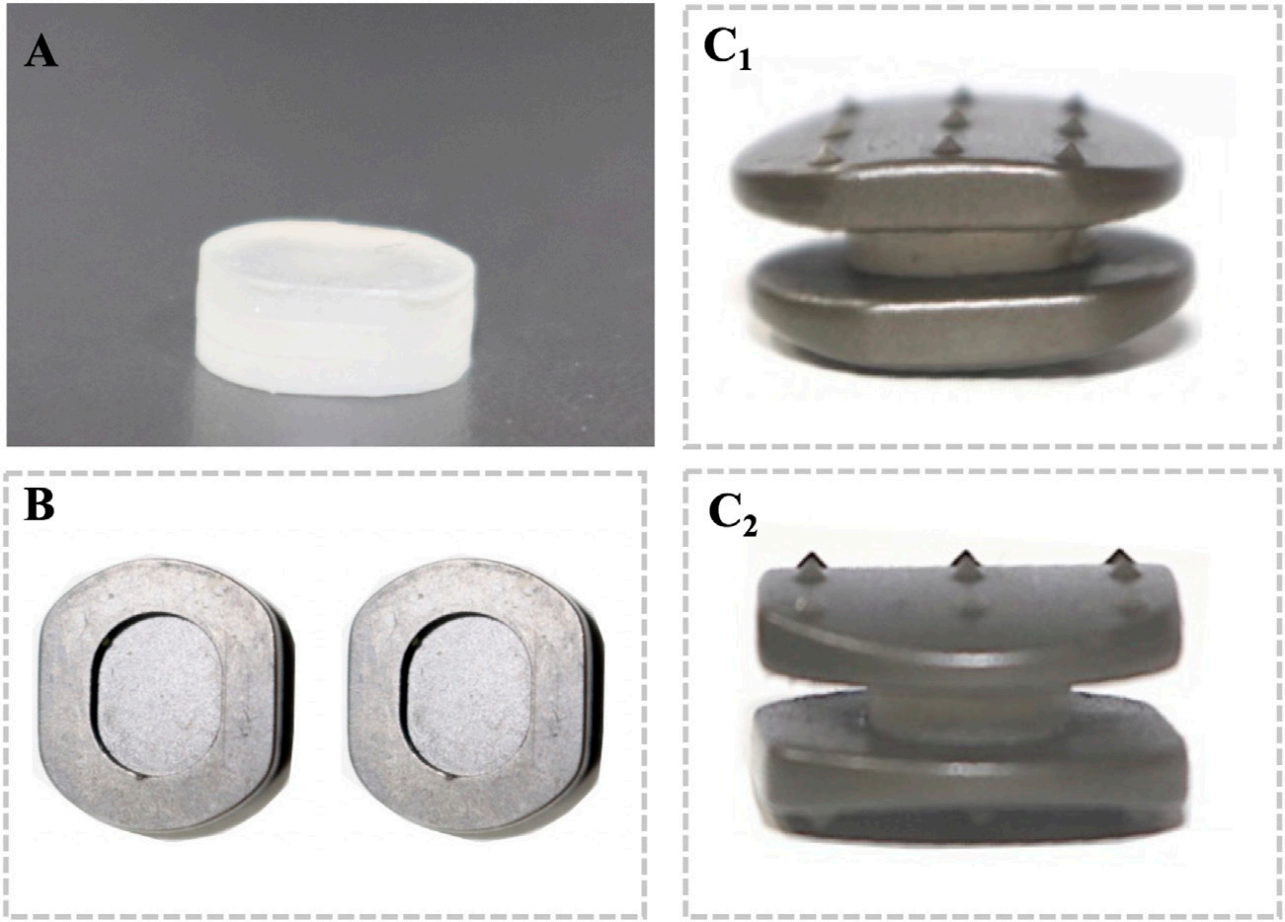
The novel cervical implant is fixed using a mortise and tenon connection between 35% hard segment polyurethane and titanium alloy (Ti-6Al-4V): **(A)** front view of the 35% hard segment polyurethane. **(B)** Top view of the internal structure of the titanium alloy plate. Coronal view **(C**_**1**_ **)** and sagittal view **(C**_**2**_ **)** of the assembled novel cervical implant.

The new prosthesis consists of an elastomeric structure with cushioning properties and a titanium alloy (Ti-6Al-4V) and they were fixed by using a mortise and tenon connection (Figure 2). Among them, the elastomeric structure was synthesized by regulating the proportion of the hard segments and soft segments of polyurethane (PU). The surface of the titanium alloy plate was subjected to microarc oxidation to further improve its strength and wearability. We developed this novel cervical implant in cooperation with Double Medical Technology Inc. (Ticker Symbol: SHE:002,901). Beside, the Zero-P VA implant was selected for ACDF group and Pretic-I artificial cervical disc was selected for ACDR group, respectively.

### Three-dimensional motion testing

**FIGURE 3:**
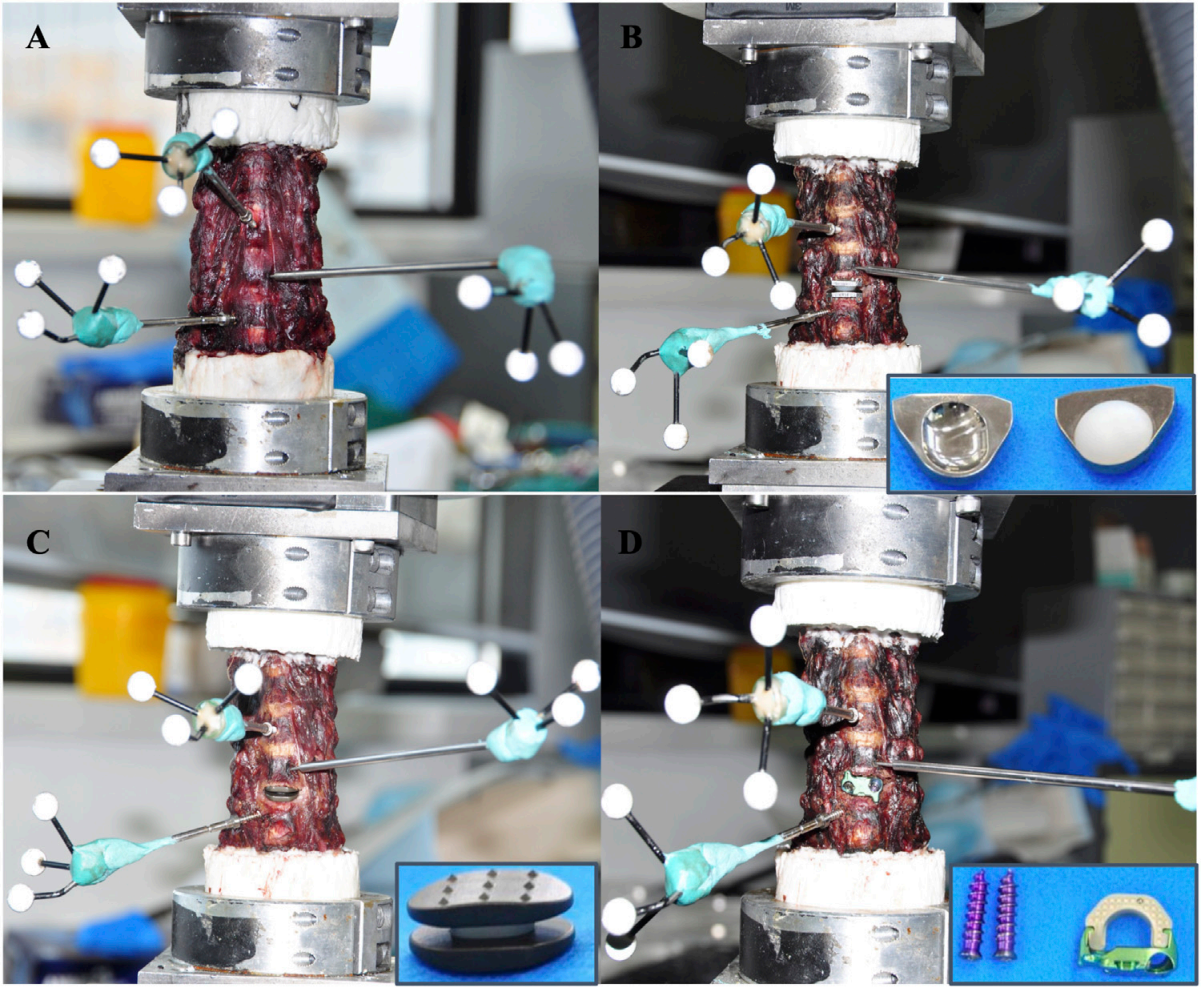
Motion capture markers of the optical tracking system were inserted into the vertebral bodies of C4–C6. **(A)** Intact group. **(B)** The Pretic-I artificial cervical disc was selected for ACDR group. **(C)** The novel cervical implant group. **(D)** The Zero-P VA implant was selected for ACDF group.

Before the biomechanical testing, the proximal (C2) and distal (C7) ends of each specimen were embedded in polymethylmethacrylate in cylindrical aluminium fixtures (the C7 vertebral segment was reinforced by partially inserting three perpendicular screws). Motion capture markers of the optical tracking system were inserted into the vertebral bodies of C4–C6. In addition, a six-axis spinal robot (Shanghai Sanyou Medical Co., Ltd., Shanghai, China) was used in this study (Figure 3). The moment arm attached to the proximal end of the specimen could apply an axial load and a pure moment, whereas the distal end of the specimen remained fixed to the socket of the robot. The robot was programmed to apply three continuous loading‒unloading cycles of the applied moment along each primary axis of motion to simulate flexion-extension (FE), lateral bending (LB), and axial rotation (AR). An axial preload of 75 N was administered on the C2 vertebra to simulate head weight (Rong et al., 2017; Wu et al., 2019; Wang et al., 2021; Wo et al., 2021; Lu et al., 2022; Zhang et al., 2023). All of the specimens were subjected to three cycles of FE, LB, and AR under a nondestructive pure moment of ±2.0 N·m, and the data of the third cycle were used for the analysis (Zheng et al., 2018). An optical tracking system (OptiTrack, NaturalPoint Inc., United States) was used to evaluate the ROM of the C4/C5, C5/C6, and C6/C7 segments. During the biomechanical tests, all of the specimens were moistened with normal saline to prevent desiccation.

### Facet joint and disc stress testing

**FIGURE 4:**
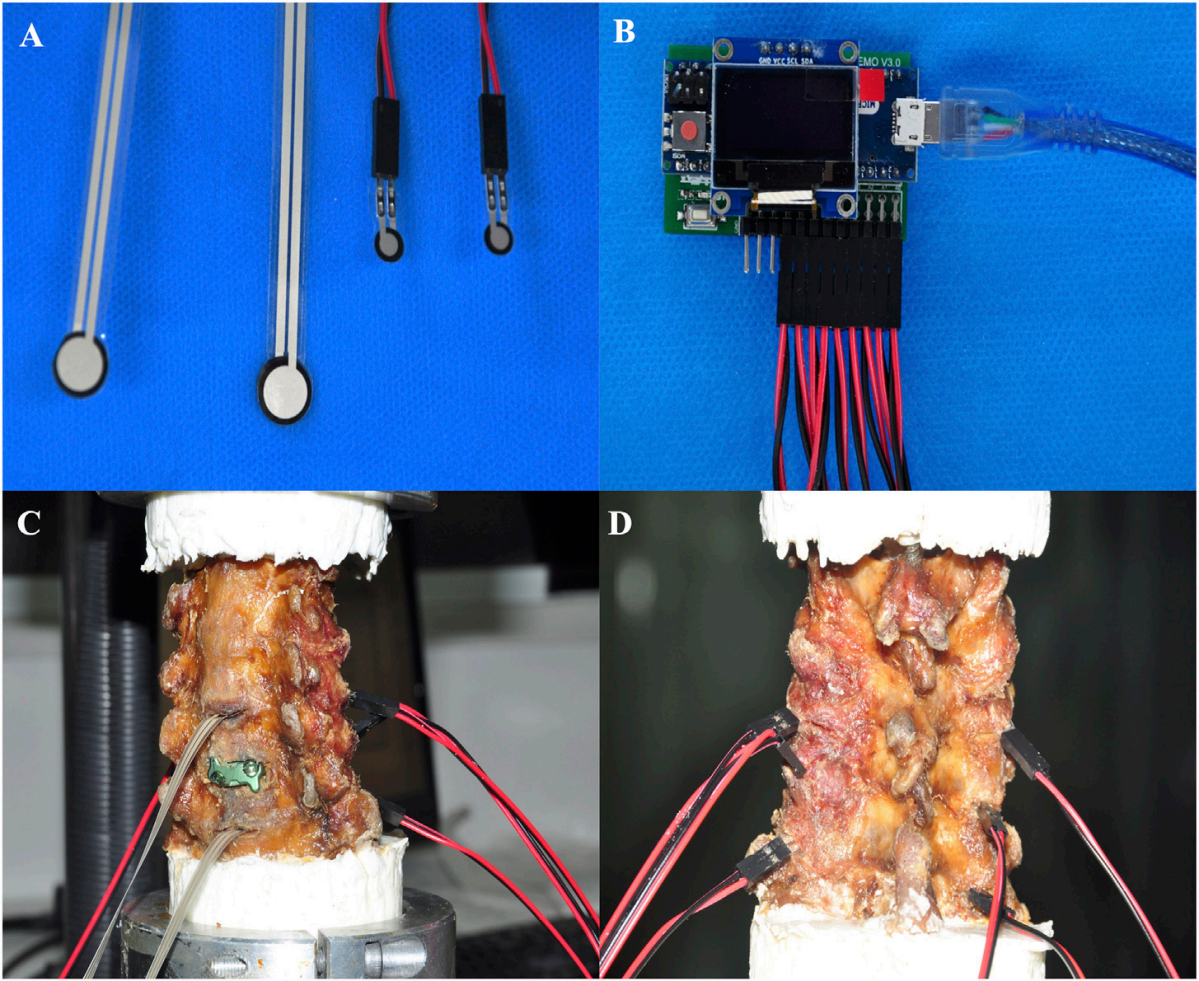
Micro pressure sensor was used to record the Maximumfacet joint pressure (FJP),maximumintradiscal pressure (IDP) at the adjacent segments. **(A)** Sensor head. **(B)** Displays. Oblique view **(C)** and posterior view **(D)** of the measurement process.

To analyse the effects of different cervical implants on the stress of the facet joints and discs at the upper and lower adjacent levels, we incised the bilateral facet joint capsules and intervertebral discs at the C4-5 and C6-7 segments. The home-built micro pressure sensors were inserted into the joint gaps and intervertebral spaces (Figure 4). The stress was recorded under 6 directions (flexion–extension, right and left axial rotations, and right and left lateral bending) (Cripton et al., 2001; Patel et al., 2017).

### Statistical analysis

We performed statistical analysis with SPSS software (version 25.0, IBM Corp). Categorical variables are summarized as percentages, and continuous variables are summarized as the mean ± standard deviation (SD). Statistical significance between two groups for ROM, facet joint pressure (FCF), maximum intradiscal pressure (IDP) was evaluated using the independent *t*-test and chi-square analysis, and one-way analysis of variance (ANOVA) was used to evaluate statistical significance among three or more groups. A two-sided *p*-value of <0.05 was considered statistically significant.

## Results

### Range of motion

**FIGURE 5:**
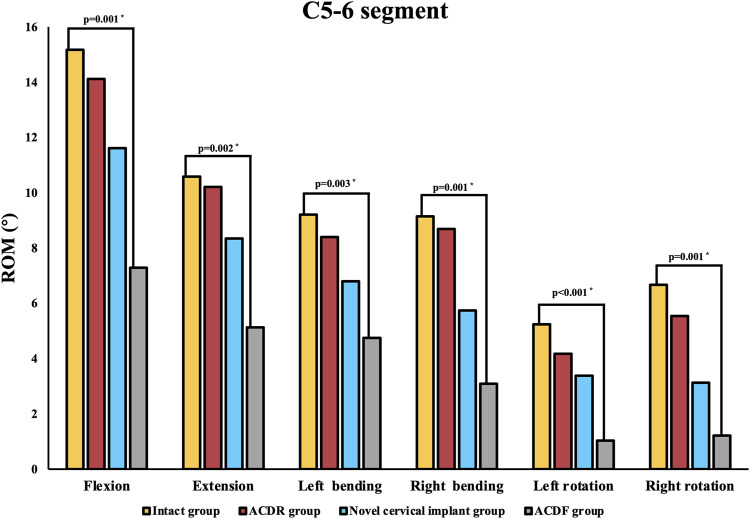
The range of motion (ROM) of each group at the C5–6 level in extension, lateral bending, and axial rotation.

**FIGURE 6:**
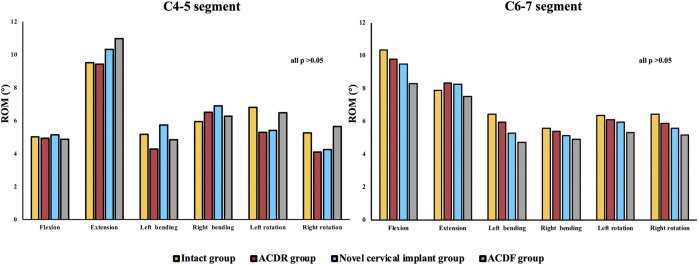
The range of motion (ROM) of each group at the C4–5 and C6–7 level in extension, lateral bending, and axial rotation.

The results are shown in Figure 5. At the operated segment, the flexion was 15.17° ± 0.88° in the intact group, 14.12° ± 1.20° in the ACDR group, 11.61° ± 1.58° in the novel cervical implant group and 7.29° ± 1.85° in the ACDF group (*p* = 0.001). The extension was 10.58° ± 1.45° in the intact group, 10.21° ± 1.34° in the ACDR group, 8.34° ± 0.69° in the novel cervical implant group and 5.13° ± 1.23° in the ACDF group (*p* = 0.002). The left bending was 9.21° ± 1.09° in the intact group, 8.40° ± 1.91° in the ACDR group, 6.80° ± 0.95° in the novel cervical implant group and 3.13° ± 1.45° in the ACDF group (*p* = 0.003). The right bending was 9.15° ± 1.39° in the intact group, 8.69° ± 1.09° in the ACDR group, 5.74° ± 0.99° in the novel cervical implant group and 3.09° ± 0.99° in the ACDF group (*p* = 0.001). The left rotation was 5.24° ± 1.04° in the intact group, 4.17° ± 0.77° in the ACDR group, 3.38° ± 0.37° in the novel cervical implant group and 1.03° ± 0.17° in the ACDF group (*p* < 0.001). The right rotation was 6.67° ± 1.34° in the intact group, 5.54° ± 1.11° in the ACDR group, 3.13° ± 0.91° in the novel cervical implant group and 1.22° ± 0.59° in the ACDF group (*p* = 0.001). Compared with the intact group, the ACDR group essentially retained the ROM of the C5/6 segment. The ROM of the C5/6 segment in the novel cervical implant group was significantly different from those values in the ACDR group and the ACDF group (*p* < 0.05). Furthermore, the ROM of the C5/6 segment in the ACDF group was lower than those values in the other groups in all directions (*p* < 0.05). Besides, There were no significant differences in the ROM of C4/5 and C6/7 between the groups (*p* > 0.05, Figure 6).

#### Maximum facet joint pressure at adjacent segments

**FIGURE 7:**
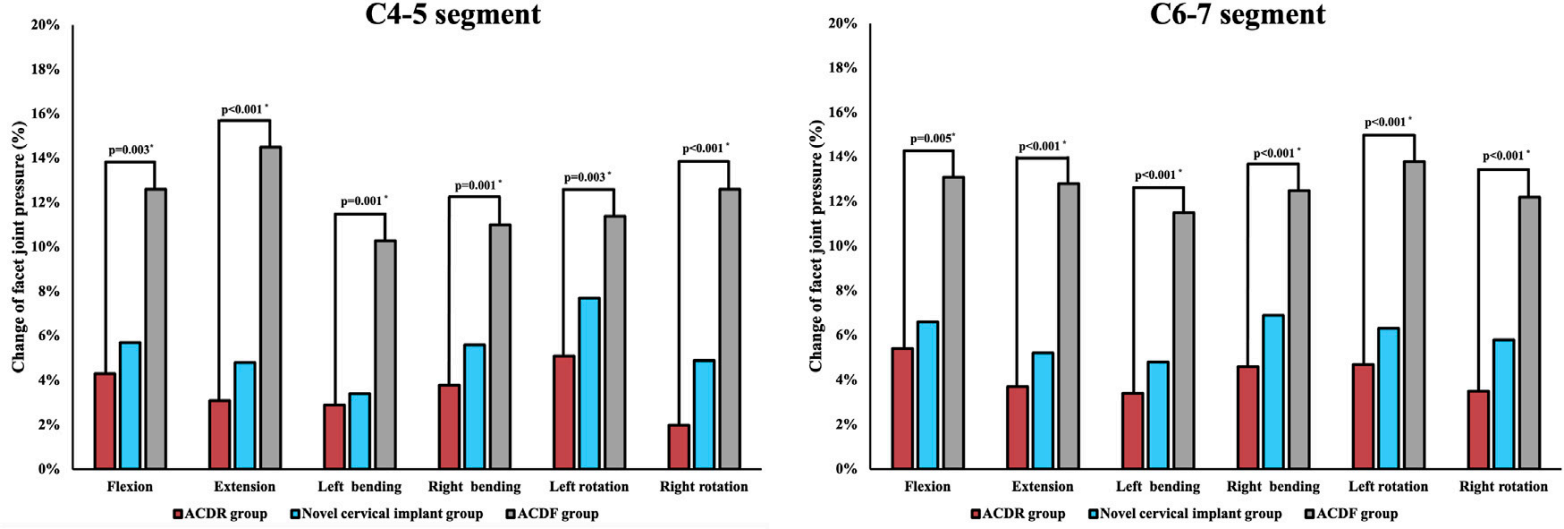
Maximum facet joint pressure (FJP) of each group at adjacent levels in extension, lateral bending, and axial rotation.

The results of maximum facet joint pressure were shown in Figure 7. Compared with the intact group, the maximum facet joint pressure of the C4/5 and C6/7 segments in the other groups increased to varying degrees. The maximum facet joint pressure at the C4/5 and C6/7 segments were increased by 5.1% and 5.4% in the ACDR group, 5.7% and 7.7% in the novel cervical implant group and 14.5% and 13.8% in the ACDF group, respectively. The maximum facet joint pressure at the C4/5 and C6/7 segments in the ACDF group were significantly higher than those values in the other groups, and there were no differences in the other groups.

#### Maximum intradiscal pressure at adjacent segments

**FIGURE 8:**
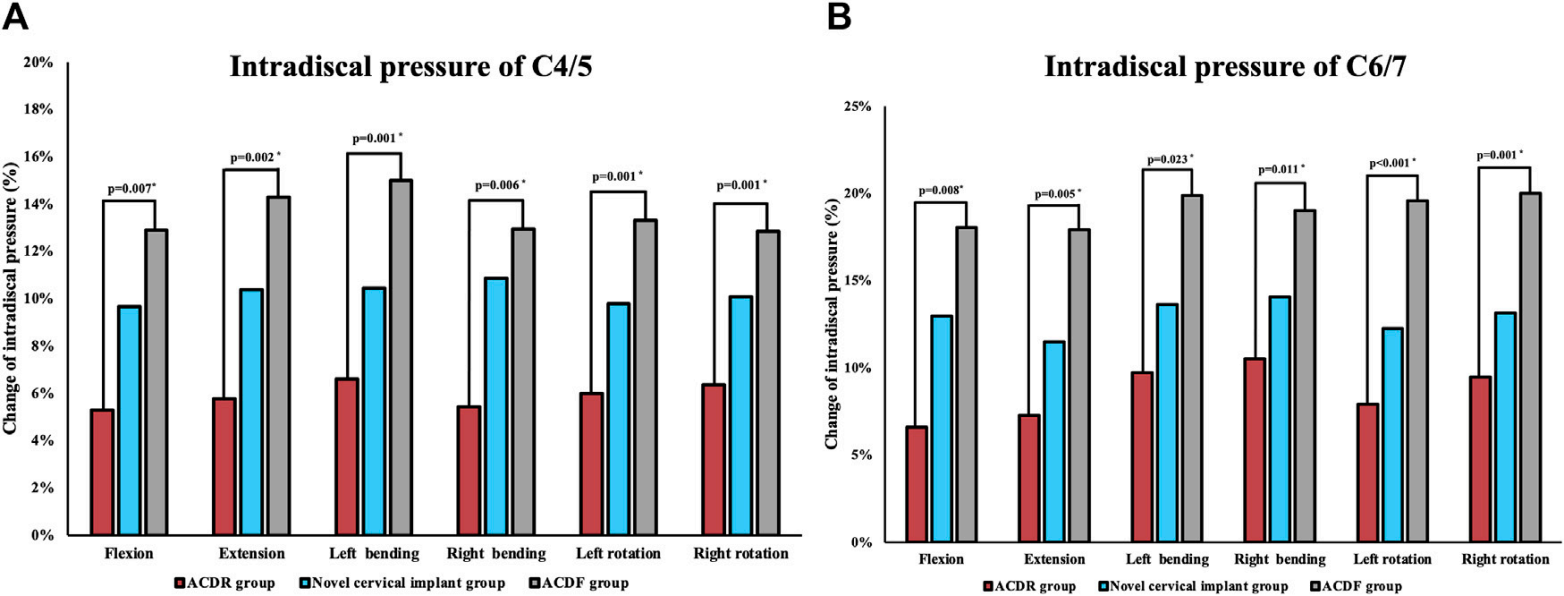
Maximum intradiscal pressure (IDP) of each group at C4/5 **(A)** and C6/7 **(B)** levels in extension, lateral bending and axial rotation.

The results of maximum intradiscal pressure (IDP) measures at uperior adjacent (C4/5), and inferior adjacent (C6/7) segments are shown in Figure 8. The intradiscal pressure at the adjacent levels in all groups was increased when compared with the intact model. The maximum increase of IDP measures was noted at the inferior adjacent (C6/7) segments under all motions in all models. The maximum intradiscal pressure at the C4/5 and C6/7 segments were increased by 6.60% and 10.51% in the ACDR group, 10.86% and 17.16% in the novel cervical implant group and 15% and 25.02% in the ACDF group, respectively. The maximum f IDP at the C4/5 and C6/7 segments in the ACDF group were significantly higher than those values in the other groups.

## Discussion

It is known that overloading at the adjacent segments caused by fusion is known to contribute to adjacent segment degeneration (Virk et al., 2014; Gornet et al., 2015). It was reported that at 7-year follow-up, the rate of adjacent-level reoperation was only 4.3% in the CDA group versus 10.8% in the ACDF cohort (Badhiwala et al., 2020b). Cervical disc replacement is a successful and promising nonfusion technique aimed at restoring normal articular motion and spine kinematics. However, the surgical indications for ACDR are particularly narrow and patients suitable for cervical disc replacement are limited. Thus, the development of a novel cervical implant that can be used by a majority of patients while reducing adjacent segment stress is of great clinical value.

The role of biomechanics and load-sharing in optimizing cervical spine surgery is not well defined. Biomechanically, the cushioning structure used in this study is unique in that it facilitates continuous load-sharing through elastic deformation and absorb interface concussion. The elastomer is fabricated from 35% hard segment polyurethane (not a low stiffness polymer) which allows true elastic deformation and continuous load-sharing through the entire range of motion of the cervical spine. Polyurethane exhibit special absorbed compressional energy performance when subjected to mechanical impact and compression, providing them with certain advantages in mimicking the cushioning and shock absorption functions of the normal intervertebral disc. Gonzalez et al. (Gonzalez Alvarez et al., 2019) also reported that elastomeric lumbar disc replacement with PU could excellently mimic the axial compliance of the spine.

There are also different dynamic stabilization devices available in the literature (Schmoelz et al., 2003; Niosi et al., 2006; Wilke et al., 2006; Schulte et al., 2008; Zhang et al., 2017). These dynamic stabilization devices functionally filled the gap between simple decompression and total rigid fixation and have achieved initial successes, and more efforts should be devoted to a broader range of applications. Ledet et al. (Ledet et al., 2018) developed a novel continuously load-sharing anterior cervical spinal plate and found that load-sharing through elastic micro-motion accelerates bone formation in the goat ACDF model. Unfortunately, the utilization of the anterior plate significantly increases the risk of dysphagia after surgery (Tortolani et al., 2006; Huang et al., 2020). In contrast, this prepared novel implant consisted of a zero profile cage combined with an individualized 3D titanium alloy plate, and this design feature could contribute to better maintenance of cervical lordosis and decrease implant-related complications.

Zhao et al. (Zhao and Yuan, 2019c) designed an innovative cervical prosthesis featuring a ball-in-socket joint structureand compared the biomechanical performance with Prestige LP artificial cervical disc and cage internal fixation using eighteen fresh-frozen human cadaveric cervical spines (C2–C7). The findings of their study demonstrated that both the novel cervical prosthesis and Prestige LP were more effective in preserving the normal range of motion in the cervical spine at the operated level and maintaining facet joint force at the adjacent level compared to cage internal fixation. Similarly, our *in vitro* investigation revealed no significant disparities in flexion-extension, lateral bending, and axial rotation between the artificial cervical disc and the novel cervical prosthesis within the C4-C5, C5-C6, and C6-C7 segments. However, the biomechanical performance of their implant was attained via the ball-in-socket joint structure, while the novel implant was prepared by facilitating continuous load-sharing through elastic deformation. To date, many studies mainly have primarily concentrated on examining the impact of novel cervical prosthesis on cervical ROM mechanics and intradiscal pressure (Cunningham et al., 2010; Demetropoulos et al., 2010; Yu et al., 2016; Lou et al., 2018), with only a limited number of studies exploring its influence on facet contact force. In fact, the facet joint plays an important role in maintaining the normal biomechanical properties of the cervical spine. According to the report, facet joints not only contribute to cervical motor function but also support approximately 30% of the load exerted on the cervical vertebrae (Pal and Sherk, 1988). Aberrant alterations in facet joint pressure may give rise to facet joint degeneration, pain, and neurological manifestations. In the present study, we performed incisions on the bilateral facet joint capsules and subsequently inserted micro pressure sensors into the joint gaps located at the C4-5 and C6-7 segments. This enabled the computer to record the distribution of stress, a method that proves to be more direct and accurate compared to alternative approaches for measuring facet joint force (Zhao and Yuan, 2019c). In our study, it was observed that the ACDF group exhibited significantly elevated maximum facet joint pressure at the C4/5 and C6/7 segments compared to the other groups. Conversely, no significant differences in maximum facet joint pressure were observed among the remaining groups.The findings of our study align with prior *in vitro* investigations, indicating that the facet joint pressure associated with ACDF was notably higher compared to the corresponding values observed in the ACDR group (Stieber et al., 2011b; Jaumard et al., 2013). Hence, in comparison to ACDF, our innovative prosthesis and artificial cervical disc exhibit superior capability in preserving the facet joint force and retarding the degeneration of the facet joint.

There exist certain limitations in this study. Primarily, the study was constrained by a scarcity of available cadavers, resulting in a relatively small sample size. Consequently, in order to mitigate potential measurement errors, the measurements were conducted thrice and the resultant average value was deemed as the definitive value. Furthermore, the C2–C7 model utilized in this study consisted of a healthy cadaveric cervical spine as opposed to a degenerative cervical spine. Additionally, the analysis focused solely on the implantation of C5/6, which is the most frequently affected single-level ACDF. In addition, the inability to replicate the postoperative state of complete bone fusion in fresh-frozen human cadaveric cervical spines limits the model’s ability to accurately reflect real-world clinical situations. Consequently, a finite element analysis can be employed to assess the long-term biomechanical performance by establishing tied contact conditions at the implant-endplate and screw-bone interfaces to simulate a fully fused state. Besides, the osteogenic potential of the novel cervical implant remains unobservable in the present cadaveric study. Consequently, it is imperative to conduct animal experiments incorporating micro CT scanning and histological analysis to assess postoperative bone formation. Another major limitation was that we are unable to analyzed the clinical efficacy and implant-related complication of this novel cervical implant in this biomechanical study. Therefore, we hope future studies, especially prospective randomized controlled studies, can answer these questions after the clinical usage of this invention.

## Conclusion

While the newly developed elastically deformable cervical implant does not completely maintain range of motion (ROM) like the artificial cervical disc, it surpasses the fusion device with regards to biomechanical attributes. After further refinement, this novel implant may be suitable for patients who are prone to severe adjacent segment degeneration after fusion surgery but no indication for artificial cervical disc surgery.

## Data Availability

The raw data supporting the conclusion of this article will be made available by the authors, without undue reservation.
